# Factors associated with ethnical disparity in overall survival for patients with hepatocellular carcinoma

**DOI:** 10.18632/oncotarget.14771

**Published:** 2017-01-20

**Authors:** Juan Li, Bettina E Hansen, Maikel P. Peppelenbosch, Robert. A. De Man, Qiuwei Pan, Dave Sprengers

**Affiliations:** ^1^ Department of Gastroenterology and Hepatology, Erasmus University Medical Center, Rotterdam, The Netherlands

**Keywords:** hepatocellular carcinoma, ethnicity, overall survival, survival disparity, race

## Abstract

Hepatocellular carcinoma (HCC) is an important cause of cancer-related death worldwide. Ethnical disparity in overall survival has been demonstrated for HCC patients in the United States (U.S.). We aimed to evaluate the contributors to this survival disparity. The SEER database was used to identify HCC patients from 2004 to 2012. Kaplan-Meier curves and Cox proportional hazard models were used to evaluate overall survival by ethnicity and the contributors to ethnical survival disparity. A total of 33 062 patients were included: 15 986 Non-Hispanic Whites, 6535 Hispanic Whites, 4842 African Americans, and 5699 Asians. Compared to Non-Hispanic Whites, African Americans had worse survival (HR, 1.18; 95%CI, 1.14–1.23), while Asians had a better survival (HR, 0.85; 95%CI, 0.82–0.89), and Hispanic Whites had a similar survival (HR, 1.01; 95%CI, 0.97–1.05). Multivariate Cox analysis identified that tumor presentation- and treatment-related factors significantly contributed to the ethnical survival disparity. Especially, tumor size was the most important contributor (HR, 1.11; 95%CI, 1.07–1.16). There is no ethnical survival disparity in patients undergoing liver transplantation and sub-analysis of patients within the Milan criteria for liver transplantation demonstrated no significant survival disparity between African Americans and non-Hispanic Whites in transplantation adjustment analysis (HR, 1.23; 95%CI, 1.11–1.35 in non-adjustment analysis to HR, 1.05; 95%CI, 0.95–1.15 after adjustment). Finally, no important contributor to the superior overall survival in Asians was identified. In conclusion, poor tumor presentation at diagnosis, limited benefit from resection and restricted utilization of liver transplantation are important contributors to poorer survival of African Americans with HCC.

## INTRODUCTION

Hepatocellular Carcinoma (HCC) is the fifth most common malignancy and the second leading cause of cancer-related death worldwide [1]. Ethnicity has been demonstrated to be related to the prognosis of HCC patients in the United States (U.S.) [2–5]. In general, African American ethnicity is associated with the poorest overall survival rate; whereas Asian ethnicity is associated with the best overall survival [3–5]. To improve the quality of health care for HCC patients, it is important to identify the factors affecting this ethnical disparity in overall survival rates (OS), and to compare their impacts.

Several factors associated with tumor presentation at time of diagnosis, type of surgical treatment, and socioeconomic status (SES), have previously been studied with regard to ethnic disparity in OS for HCC patients. Previous literature has reported that African American patients have a more advanced, and Asian patients a less advanced tumor stage at the moment of diagnosis, compared to non-Hispanic white patients [6, 7]. Obviously, more advanced disease at time of diagnosis may affect OS. However, access to curative treatment options may also play a role. African American patients have been demonstrated to have less surgical treatment (resection or liver transplantation (LT)) than non-Hispanic white patients, and Asian patients are less likely to have transplantation but more likely to have hepatectomy than non-Hispanic white patients [6–8]. However, it should be noted that there have been inconsistent reports about treatment effects. Mathur et al [9] reported that after tumor ablation and hepatic resection, African American and Hispanic patients had the worst survival. Asian patients had better survival than white patients after ablation and similar survival after hepatectomy. After liver transplantation, there was no significant difference in survival by race/ethnicity [9]. On the other hand, several other studies have reported that African American patients have worse OS after liver transplantation than non-Hispanic white patients [3, 10]. Finally, socioeconomic status (SES) could be the potential driving factor for ethnical survival disparity as it can affect healthcare-utilization (early detection, treatment, and post-treatment quality of life) in cancer patients [11]. However, several studies have found that SES does not explain ethnic disparity in OS for HCC patients [11, 12].

Inconsistencies in study results might be due to differences between the populations and study designs. Nevertheless, there are many socioeconomic and tumor- and treatment-related factors that may impact racial disparity in survival of HCC patients more or less, and as far as we know their influence on OS has not been compared [4]. In this study we describe the relative contributions of these factors to the ethnical disparity in OS for HCC patients, using The Surveillance, Epidemiology, and End Results (SEER) database.

## RESULTS

### Population

Based on inclusion criteria, this study included a total of 33 062 patients who were diagnosed with HCC from 2004 to 2012 (Supplementary Figure 1). The population characteristics are summarized in Table 1. Among them, 15 986 (48%) were Non-Hispanic Whites, 6535 (20%) were Hispanic Whites, 4842 (15%) were African Americans, and 5699 (17%) were Asians (Asian or Pacific Islander). Although not statistically significant, in comparison to other ethnicities, Asians had a higher average age (mean age: 63 years, IQR:55–73) than Non-Hispanic Whites (mean age: 62; IQR: 55–72) and African Americans (mean age: 59; IQR: 54–64). Additionally, African Americans were more likely to be diagnosed with large tumor (tumor size > 5 cm) than Non-Hispanic Whites, and *vice versa*. For example, among African Americans 38% had large tumor and 17% had small tumor; among Non-Hispanic Whites 34% had large tumor and 22% had small tumor (*p* < .0001).

**Table 1:** Characteristics of all patients by ethnicity

		Ethnicity				
Characteristics	Total	Non-Hispanic White	Hispanic White	African American	Asian	*P* Value
Patients						
No. %	33 062	15 986 (48)	6535 (20)	4842 (15)	5699 (17)	
Age						< .0001^a^
Mean [SD],y	63 [12]	63 [11]	62 [12]	60 [10]	63 [13]	
Median [IQR],y	61 [55–71]	62 [55–72]	60 [53–70]	59 [54–64]	63 [55–73]	
Gender (%)						< .0001^b^
Male	25 728 (78)	12 698 (79)	5061 (77)	3779 (78)	4190 (74)	
Female	7334 (22)	3288 (21)	1474 (23)	1063 (22)	1509 (27)	
Marital status (%)						< .0001^a^
Married	16937 (51)	8151 (51)	3285 (50)	1607 (33)	3894 (68)	
Unmarried	14775 (45)	7158 (45)	3003 (46)	2999 (62)	1615 (28)	
Unknown	1350 (4)	677 (4)	247 (4)	236 (5)	190 (3)	
Education (%)^c^						< .0001^b^
Mean [SD]	16 [6]	15 [6]	19 [6]	16 [5]	16 [5]	
Median [IQR]	15 [12–22]	14 [11–20]	20 [14–23]	15 [12–18]	14 [12–23]	
Poverty (%)						< .0001^a^
Mean [SD]	16 [5]	15 [5]	17 [4]	18 [6]	14 [4]	
Median [IQR]	16 [12–18]	14 [12–18]	18 [13–18]	18 [13–23]	13 [10–18]	
Income (%)						< .0001^a^
Mean [SD]	59619 [14401]	58 600 [14690]	59478 [12533]	53924 [13423]	67477 [13132]	
Median [IQR]	55910 [50588–69710]	56490 [48260–66520]	55910 [54090–62960]	55060 [41180–61260]	67180 [55910–75600]	
Residence (%)						< .0001^b^
Rural	2592 (8)	1957 (12)	255 (4)	265 (5)	115 (2)	
Urban	30470 (92)	14029 (88)	6280 (96)	4577 (95)	5584 (98)	
Lesion number (%)						.002^b^
Single	32015 (97)	15438 (97)	6373 (98)	4697 (97)	5507 (97)	
Multiple	1047 (3)	548 (3)	162 (2)	145 (3)	192 (3)	
Grade (%)						< .0001^b^
Well differentiated	4325 (13)	2209 (14)	883 (14)	598 (12)	635 (11)	
Moderately differentiated	5377 (16)	2664 (17)	896 (14)	797 (16)	1020 (18)	
Poorly differentiated	2854 (9)	1358 (8)	466 (7)	438 (9)	592 (10)	
Undifferentiated	281 (1)	140 (1)	43 (1)	39 (1)	59 (1)	
Unknown	20225 (61)	9615 (60)	4247 (65)	2970 (61)	3393 (60)	
Stage (%)						< .0001^b^
Localized	16143 (49)	7822 (49)	3304 (51)	2175 (45)	2842 (50)	
Regional	9618 (29)	4573 (29)	1849 (28)	1473 (30)	1723 (30)	
Distant	5201 (16)	2466 (15)	984 (15)	906 (19)	845 (15)	
Unstaged	2100 (6)	1125 (7)	398 (6)	288 (6)	289 (5)	
Tumor size (cm),%						< .0001^b^
< 3	6693 (20)	3499 (22)	1335 (20)	808 (17)	1051 (18)	
3–5	7519 (23)	3613 (23)	1601 (24)	1030 (21)	1275 (22)	
> 5	12184 (37)	5497 (34)	2370 (36)	1847 (38)	2470 (43)	
Unknown	6666 (20)	3377 (21)	1229 (19)	1157 (24)	903 (16)	
Lymph node involvement (%)						< .0001^b^
No lymph node	25833 (78)	12380 (77)	5091 (78)	3753 (78)	4609 (81)	
Lymph node	2272 (7)	1239 (8)	351 (5)	401 (8)	281 (5)	
Unknown	4957 (15)	2367 (15)	1093 (17)	688 (14)	809 (14)	
Vascular Invasion (%)						< .0001^b^
No Vascular Invasion	16749 (51)	8187 (51)	3412 (52)	2297 (47)	2853 (50)	
Vascular Invasion	13467 (41)	6269 (39)	2569 (39)	2137 (44)	2492 (44)	
Unknown	2846 (9)	1530 (10)	554 (8)	408 (8)	354 (6)	
Metastatic status (%)						< .0001^b^
No metastasis	25038 (76)	12119 (76)	4937 (76)	3579 (74)	4403 (77)	
Metastasis	5154 (16)	2469 (15)	989 (15)	882 (18)	814 (14)	
Unknown	2870 (9)	1398 (9)	609 (9)	381 (8)	482 (8)	
AFP^d^ (%)						< .0001^b^
Positive	19366 (59)	8672 (54)	3928 (60)	3221 (67)	3545 (62)	
Negative	5705 (17)	3002 (19)	1186 (18)	546 (11)	971 (17)	
Borderline	74 (0)	48 (0)	8 (0)	10 (0)	8 (0)	
Unknown	7917 (24)	4264 (27)	1413 (22)	1065 (22)	1175 (21)	
Fibrosis (%)						< .0001^b^
None to moderate fibrosis	1622 (5)	723 (5)	236 (4)	220 (5)	443 (8)	
Severe fibrosis or cirrhosis	5448 (16)	2678 (17)	1209 (19)	709 (15)	852 (15)	
Unknown	25992 (79)	12585 (79)	5090 (78)	3913 (81)	4404 (77)	
Treatment (%)						< .0001^b^
None	24646 (75)	11672 (73)	5178 (79)	3850 (80)	3946 (69)	
Tumor destruction	3102 (9)	1544 (10)	596 (9)	368 (8)	594 (10)	
Surgical resection	3002 (9)	1364 (9)	349 (5)	406 (8)	883 (15)	
Liver transplantation	2016 (6)	1221 (8)	374 (6)	178 (4)	243 (4)	
Unknown	296 (1)	185 (1)	38 (1)	40 (1)	33 (1)	

^a^ one-way ANOVA test.

^b^ Pearson Chi-Square.

^c^ Indicates the percentage of adults aged ≥ 25 years who had < 12 years of education.

^d^ AFP positive indicates AFP > 15 ng/ml.

### Ethnical disparity in overall survival in overall HCC population

**Figure 1:**
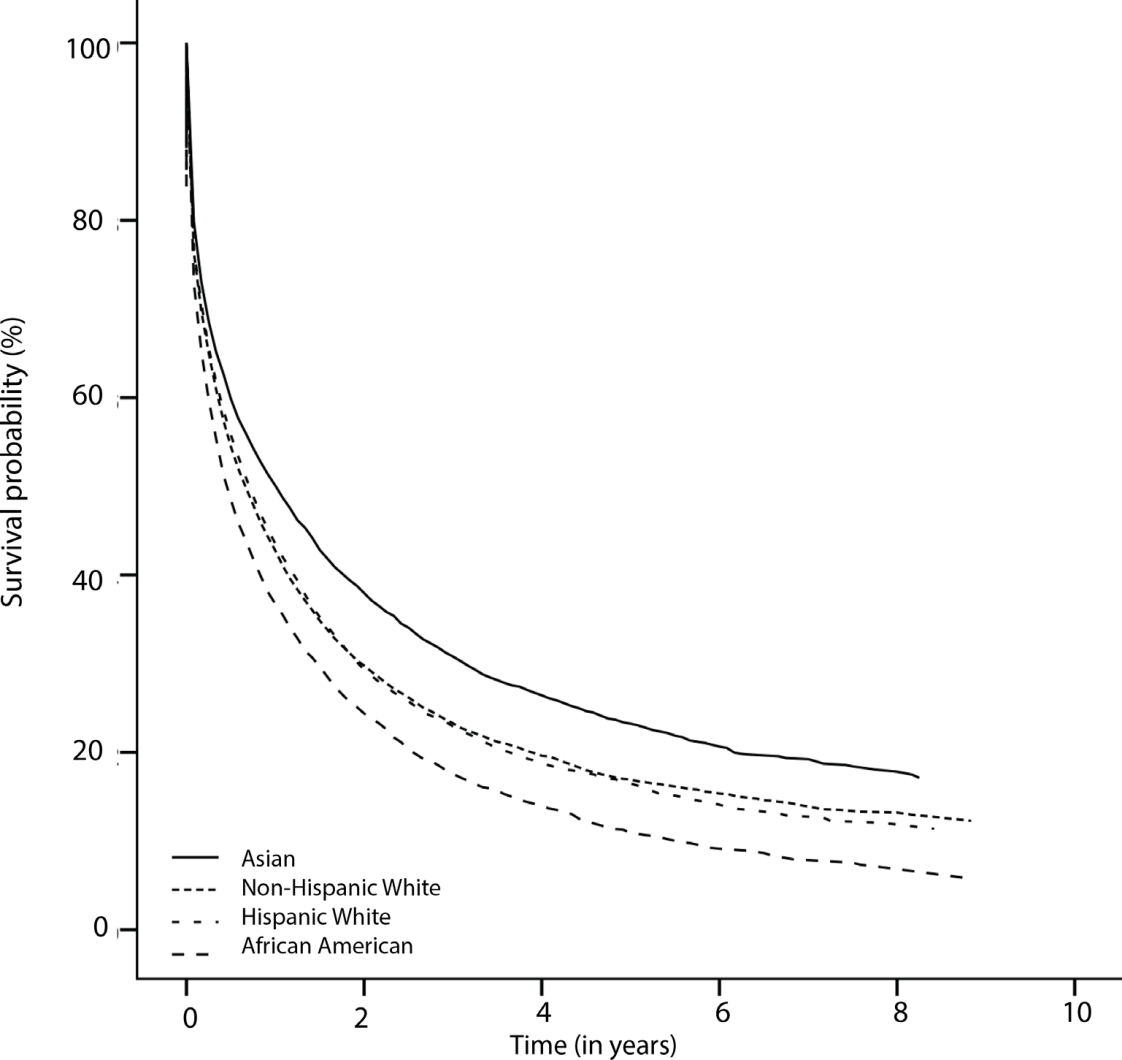
The Kaplan-Meier survival curves showing ethnical survival disparities

Figure 1 displays the overall survival (OS) rates among different ethnical populations. The median survival was 8 months (95%CI: 7.6–8.4), 9 months (95%CI: 8.4–9.6), 6 months (95%CI:5.5–6.5), and 13 months (95%CI: 12.0–14.0) for Non-Hispanic White, Hispanic White, African American, and Asian patients, respectively. 1-year and 3-year survival rates were 44% and 24%, 45% and 23%, 38% and 18%, and 51% and 31% for Non-Hispanic White, Hispanic White, African American, and Asian patients, respectively. Therefore, Hispanic White and Non-Hispanic White patients had similar survival rates. Asian patients displayed the best OS, and African American patients had the poorest OS. Specifically, there was significant “negative” survival disparity between African American and Non-Hispanic White patients (*P* < .0001), and “positive” survival disparity between Asian and White patients (*P* < .0001).

**Figure 2:**
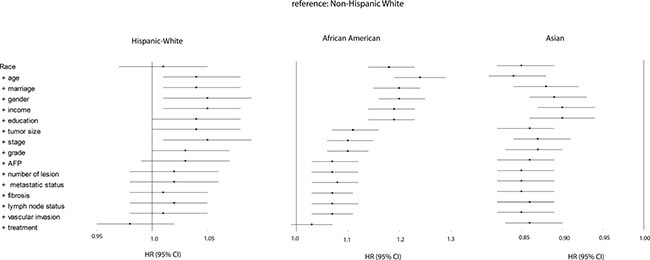
Forest plot presenting the estimated HR's of ethnicity on overall survival from multivariate Cox models for all ethnical groups (reference: Non-Hispanic White) The first HR is the crude effect followed by HR after adjustment entering covariates in a forward stepwise manner (LR): age, marriage, gender, income, education, tumor size, stage, grade, AFP, number of lesion, metastatic status, fibrosis, lymph node status, vascular invasion, and treatment. Block 1 included race, block 2 included age, gender, marital status, education, income, poverty, residence, block 3 included grade, stage, number of lesion, tumor size, lymph node status, vascular invasion, metastatic status, AFP, and fibrosis, and block 4 included treatment.

To determine the importance of several demographic-, tumor- and treatment-related factors for ethnical survival disparity, we performed multivariate analyses, and then observed the change of hazard ratios (HRs). Figure 2 shows a forest plot presenting results from multivariate Cox models for all ethnical groups in the overall population (reference: Non-Hispanic White). No significant difference was observed between Hispanic White and Non-Hispanic White both in univariate analysis (HR, 1.01; 95%CI, 0.97–1.05) and multivariate analysis (HR, 0.98; 95%CI, 0.95–1.02). However, with respect to African American patients, we noticed some remarkable changes in survival disparity in Cox models. The initial survival disparity between African American and Non-Hispanic White (HR, 1.18; 95%CI, 1.14–1.23) did not change much when we adjusted demography-related variables. However, it was affected by tumor size (HR, 1.11; 95%CI, 1.07–1.16), which indicated that the increased occurrence of large tumor in African Americans was associated with their poor survival. The other tumor-related variables that we studied did not significantly change the survival disparity any further. After additional adjustment for treatment-related factors, the significant survival disparity between African Americans and Non-Hispanic Whites became non-significant (HR, 1.03; 95%CI, 0.99–1.07). Therefore, we conclude that tumor size and treatment contributed largely to the survival disparity between African American and Non-Hispanic White patients. When comparing Non-Hispanic Whites to Asian patients, the latter population displayed a significantly better survival (HR, 0.85; 95%CI, 0.82–0.89), which remained constant from univariate analysis (HR, 0.85; 95%CI, 0.82–0.89) to multivariate analysis (HR, 0.86; 95%CI, 0.83–0.90). In other words, we did not identify the contributors to superior survival in Asian patients.

Since, for a large group of patients fibrosis scores were unavailable in the full SEER dataset, which may present a bias with respect to the survival data, we further analyzed a subset of patients for which this fibrosis score was available (*n* = 7070, characteristics in Supplementary Table 1). Supplementary Figure 3 demonstrates that this subpopulation African Americans also had a poorer survival than Non-Hispanic Whites (HR, 1.19; 95%CI, 1.08–1.31). This survival disparity in multivariate analysis was again affected by tumor size; the factor large tumor size was associated with poor survival (HR, 1.10, 95%CI, 1.00–1.22).

### Ethnical disparity in overall survival in patients stratified by treatment

**Figure 3:**
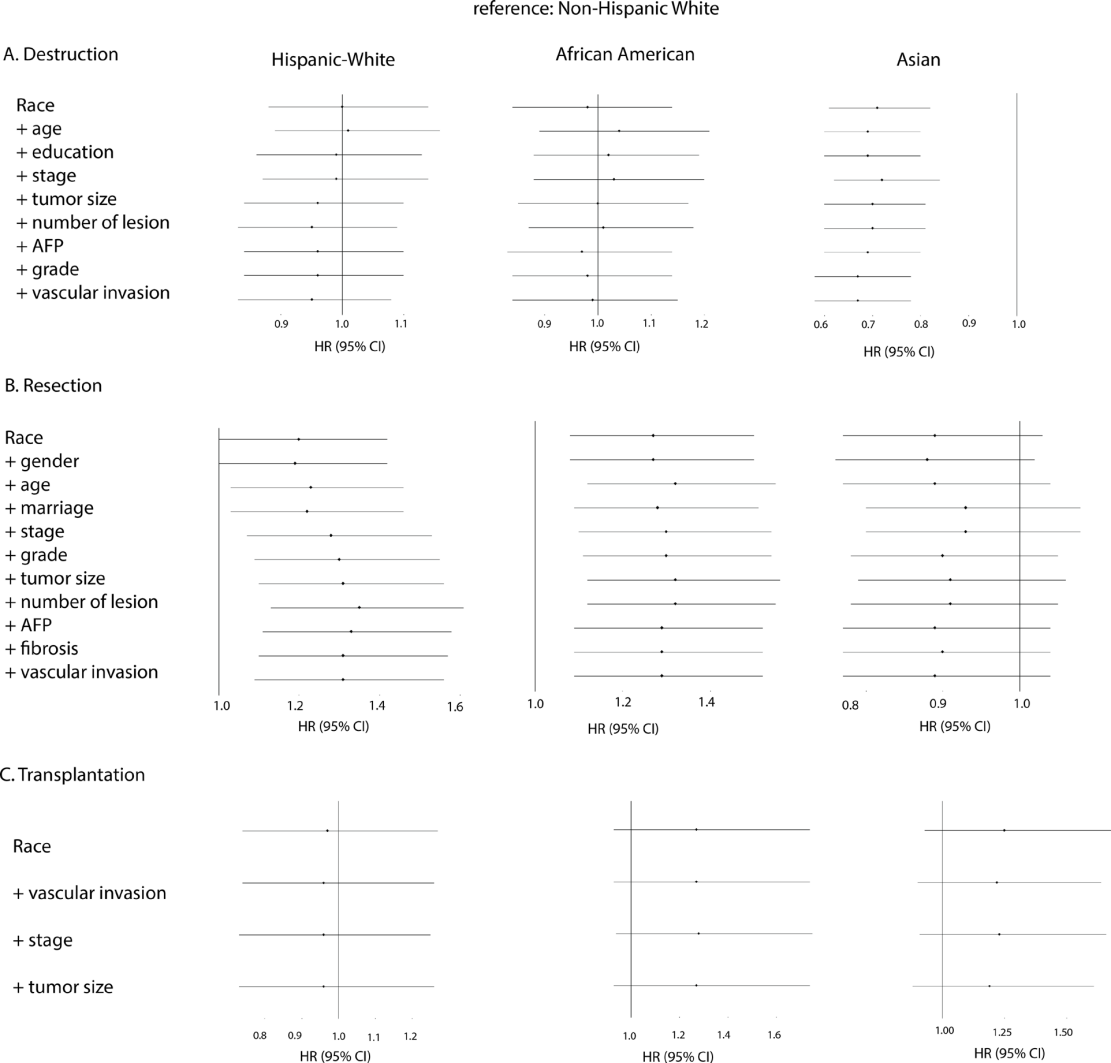
Forest plot presenting the estimated HR's of ethnicity on overall survival from multivariate Cox models for all ethnical groups stratified by treatment (reference: Non-Hispanic White) Forward stepwise method was used to study the changes of HR of ethnicity after entering the following covariates: In the stratum (**A**) Destruction: race, age, education, stage, tumor size, number of lesion, AFP, grade, and vascular invasion. In the stratum (**B**) Resection: race, gender, age, marriage, stage, grade, tumor size, number of lesion, AFP, fibrosis, and vascular invasion. And in the stratum (C) Transplantation: race, vascular invasion, stage, and tumor size.

We further explored the survival patterns among ethnicities in subgroups stratified by treatment: patients treated with tumor destruction (radiofrequent ablation / percutaneous ethanol injection (PEI) etc.) (9% of total), those that had surgical resection (9% of total), and those that have had liver transplantation (6% of total) (Table 1). As for the patients who underwent tumor destruction, both African Americans and Hispanic Whites showed non-significant survival difference compared to Non-Hispanic Whites. Asians had a much higher survival rate than Non-Hispanic Whites (HR, 0.71; 95%CI, 0.61–0.82) and no specific reason was found for this disparity (Figure 3A). For patients receiving surgical resection, we found significantly lower survival rates in Hispanic Whites (HR, 1.20; 95%CI, 1.00–1.42) and African Americans (HR, 1.27; 95%CI, 1.08–1.50) than in Non-Hispanic Whites. Demographic and tumor-related factors negatively influenced survival in Hispanic Whites but for African Americans no such contribution could be identified among the various Cox models. Of interest, after surgical resection Asians did not have significantly higher survival rates than Non-Hispanic Whites (Figure 3B). Interestingly, no significant ethnical difference in survival was detected in patients after liver transplantation (Figure 3C).

### Ethnical survival disparity in patients eligible for liver transplantation

**Figure 4:**
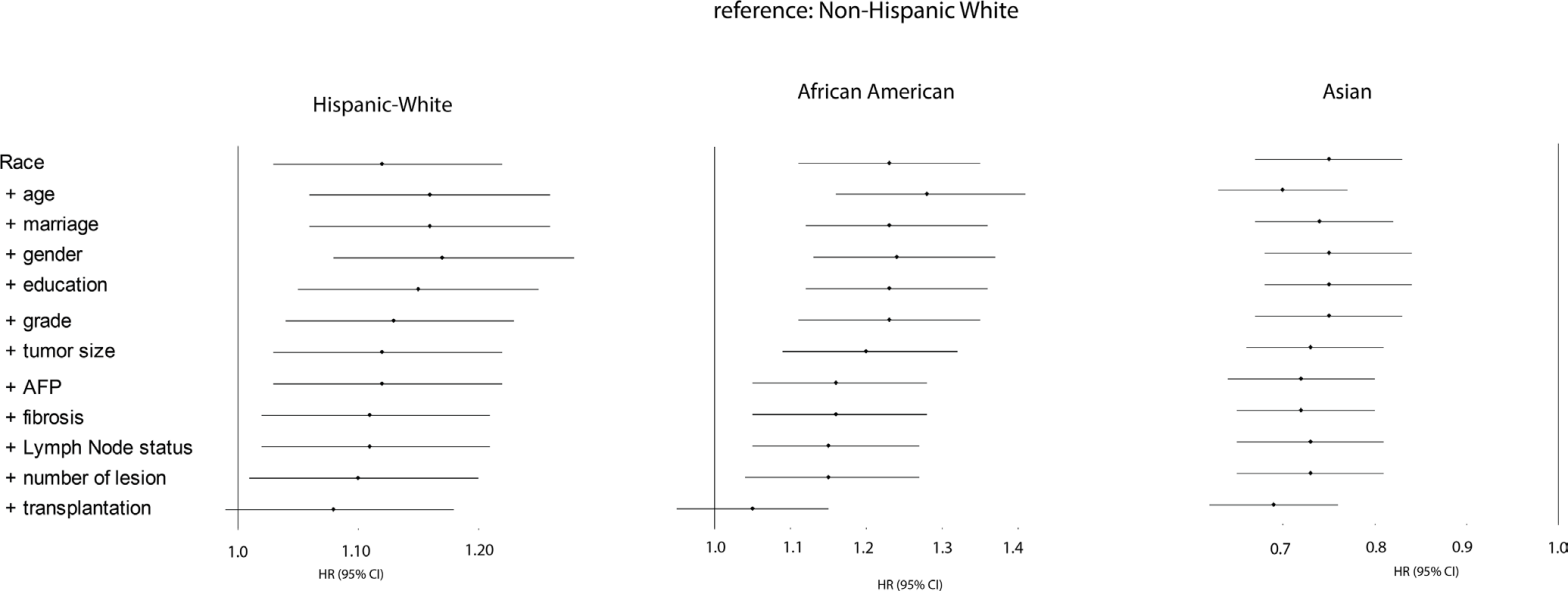
Forest plot presenting the estimated HR's of ethnicity on overall survival from multivariate Cox models for all ethnical groups who met Milan criteria (reference: Non-Hispanic white) The first HR is the crude effect followed by HR after adjustment entering covariates in a forward stepwise manner (LR): age, marriage, gender, education, grade, tumor size, AFP, fibrosis, lymph node status, number of lesion, and transplantation. Block 1 included race, block 2 included age, gender, marital status, education, income, poverty, residence, block 3 included grade, stage, number of lesion, tumor size, lymph node status, vascular invasion, metastatic status, AFP, and fibrosis, and block 4 included treatment.

To further explore the impact of liver transplantation on ethnical survival disparity, we performed survival analyses in a subgroup of patients who met the Milan criteria for liver transplantation (1 nodule ≤ 5 cm and max 3 nodules ≤ 3 cm and no signs of vascular invasion/extrahepatic spread). Table 2 describes the characteristics for those patients. The patients receiving liver transplantation accounted for 15% of total patients “within Milan”; 19% of Non-Hispanic Whites (*n* = 802), 11% of African Americans (*n* = 111), 13% of Hispanic Whites (*n* = 232), and 10% of Asians (*n* = 137) (*P* < .0001). The survival rates are displayed in Figure 4. Compared to Non-Hispanic Whites, Hispanic White patients exhibited a poorer survival (HR, 1.12; 95%CI, 1.03–1.22), which was improved when adjusting for tumor-related factors but became not significant after adjusting for liver transplantation. Also, African American patients displayed a poorer survival (HR, 1.23; 95%CI, 1.11–1.35), and their outcome was improved when adjusting tumor-related variables. But especially when transplantation status was adjusted, the survival discrepancy disappeared (HR, 1.05; 95%CI, 0.95–1.15). Finally, the superior survival remained constant for Asian patients from crude analysis to adjustment analyses.

**Table 2:** Characteristics of the patients within Milan criteria

		Ethnicity				
Characteristics	Total	Non-Hispanic White	Hispanic White	African American	Asian	*P* Value
Patients						
No. %	8 524	4290 (50)	1779 (21)	1056 (12)	1399 (16)	
Age						< .0001
Mean [SD],y	61 [10]	61 [10]	60 [10]	60 [9]	64 [11]	
Median [IQR],y	60 [54–68]	60 [55–67]	59 [53–67]	59 [55–64]	63 [55–72]	
Gender (%)						< .0001
Male	6425 (75)	3380 (79)	1319 (74)	791 (75)	935 (67)	
Female	2099 (25)	910 (21)	460 (26)	265 (25)	464 (33)	
Marital status (%)						< .0001
Married	4471 (52)	2223 (52)	896 (50)	379 (36)	973 (70)	
Unmarried	3732 (44)	1893 (44)	825 (46)	625 (59)	389 (28)	
Unknown	321 (4)	174 (4)	58 (3)	52 (5)	37 (3)	
Education						< .0001
Mean [SD]	16 [6]	15 [6]	19 [6]	16 [5]	16 [5]	
Median [IQR]	15 [12–22]	14 [11–19]	18 [14–23]	15 [12–16]	14 [13–20]	
Poverty						< .0001
Mean [SD]	15 [5]	15 [5]	16 [5]	18 [6]	14 [4]	
Median [IQR]	15 [12–18]	14 [12–18]	18 [13–18]	18 [13–21]	13 [10–18]	
Income						< .0001
Mean [SD]	60176 [14217]	58775 [14214]	60168 [12703]	54675 [13422]	68633 [13069]	
Median [IQR]	56530 [51380–72110]	56530 [48510–65590]	55910 [54090–63360]	55060 [41180–62000]	72110 [55910–75600]	
Residence (%)						< .0001
Rural	585 (7)	468 (11)	60 (3)	41 (4)	16 (1)	
Urban	7939 (93)	3822 (89)	1719 (97)	1015 (96)	1383 (99)	
Lesion number (%)						.100
Single	8309 (97)	4170 (97)	1747 (98)	1033 (98)	1359 (97)	
Multiple	215 (3)	120 (3)	32 (2)	23 (2)	40 (3)	
Grade (%)						< .0001
Well differentiated	1457 (17)	766 (18)	293 (16)	176 (17)	222 (16)	
Moderately differentiated	1582 (19)	807 (19)	270 (15)	213 (20)	292 (21)	
Poorly differentiated	461 (5)	230 (5)	79 (4)	49 (5)	103 (7)	
Undifferentiated	39 (0)	20 (0)	7 (0)	3 (0)	9 (1)	
Unknown	4985 (58)	2467 (58)	1130 (64)	615 (58)	773 (55)	
Stage (%)						.013
Localized	8228 (97)	4128 (96)	1728 (97)	1009 (96)	1363 (97)	
Regional	295 (3)	162 (4)	51 (3)	46 (4)	36 (3)	
Distant	1 (0)	0 (0)	0 (0)	1 (0)	0 (0)	
Tumor size (cm),%						< .001
< 3	4465 (52)	2348 (55)	901 (51)	524 (50)	692 (49)	
3–5	4059 (48)	1942 (45)	878 (49)	532 (50)	707 (51)	
Lymph node involvement (%)						.001
No lymph node	8173 (96)	4099 (96)	1706 (96)	1004 (95)	1364 (97)	
Lymph node	149 (2)	92 (2)	19 (1)	25 (2)	13 (1)	
Unknown	202 (2)	99 (2)	54 (3)	27 (3)	22 (2)	
Vascular Invasion (%)						NA
No Vascular Invasion	8524 (100)	4290 (100)	1779 (100)	1056 (100)	1399 (100)	
Metastatic status (%)						NA
No metastasis	8524 (100)	4290 (100)	1779 (100)	1056 (100)	1399 (100)	
AFP (%)						< .0001
Positive	4629 (54)	2126 (50)	985 (55)	682 (65)	836 (60)	
Negative	2176 (26)	1196 (28)	482 (27)	175 (17)	323 (23)	
Borderline	21 (0)	15 (0)	2 (0)	2 (0)	2 (0)	
Unknown	1698 (20)	953 (22)	310 (17)	197 (19)	238 (17)	
Fibrosis (%)						< .0001
None to moderate fibrosis	523 (6)	253 (6)	59 (3)	68 (6)	143 (10)	
Severe fibrosis or cirrhosis	2139 (25)	1081 (25)	469 (26)	261 (25)	328 (23)	
Unknown	5862 (69)	2956 (69)	1251 (70)	727 (69)	928 (66)	
Therapy (%)						< .0001
None	4478 (53)	2167 (51)	1103 (62)	604 (57)	604 (43)	
Tumor destruction	1746 (20)	864 (20)	320 (18)	202 (19)	360 (26)	
Surgical resection	980 (11)	432 (10)	120 (7)	133 (13)	295 (21)	
Liver transplantation	1282 (15)	802 (19)	232 (13)	111 (11)	137 (10)	
Unknown	38 (0)	25 (1)	4 (0)	6 (1)	3 (0)	

## DISCUSSION

Since HCC related mortality continues to increase in the US, the ethnical disparities in overall survival has attracted attention [13]. Many efforts have been devoted to exploring the reasons behind this phenomenon [4, 14] for a better understanding of its contributors, which shall help us to determine which interventions could reduce this disparity.

We have confirmed the ethnical survival disparity in overall survival that has previously been reported by others [3–6, 9]. We demonstrated how tumor-related and treatment-related factors contribute strongly to survival disparity between African American and Non-Hispanic White patients.

As demonstrated in previous studies, compared to non-Hispanic Whites we found a poor survival in African Americans and a good survival in Asian patients [4, 7, 9, 12, 15, 16]. Our results demonstrate that increased presence of large tumor size was associated with poor survival in African Americans. Tumor size is considered as an important prognostic determinant in several HCC staging systems such as the TNM classification [17], the Barcelona Clinic Liver Cancer (BCLC) staging system [18], and the Hong Kong Liver Cancer (HKLC) classification [19]. Although previous studies have reported the significant differences in tumor size in HCC patients stratified by race and proposed it as a predictor of prognosis for HCC patients [4, 7, 9, 12, 15, 16], our study has demonstrated that tumor presentation indeed is the dominant contributor to the poor OS of African Americans. Such a clear dominant factor could not be demonstrated for Hispanic-Whites and Asians. In the former population demographic factors contributed to OS to some extent, but again presentation-related factors were shown to be the dominant contributors. Stage of liver disease as represented by fibrosis score did not significantly impact the observed survival disparities, as in a sub-analysis of patients of which these data were available tumor size remained a major confounder.

Consistent with previously studies, we found superior OS in Asians. Marital status affected HRs to some extent, and in our population Asians showed the highest percentage of marriage: up to 70% (*P* < .0001). Multiple studies have shown that being married is associated with more favorable survival for various cancer types [20–23] and this also appears to be the case in Asian HCC patients.

Treatment related factors also contributed to ethnical survival differences. As reported by others [9] African Americans displayed the poorest response to resection. We cannot explain this finding based on our data. African Americans were previously reported to have a longer waiting time period before surgery [12], which may affect the severity of their liver disease and consequently the chance on complications after surgery. However, this information was not available for us to study.

We speculate that there is an impact of the etiology of liver disease on both the observed overall survival disparities and the discrepancies found in relation to treatment modality. The cause of liver disease in the majority of Asian HCC patients is chronic HBV infection; whereas in African Americans and non-Hispanic Whites chronic HCV infection, non-alcoholic fatty liver disease (NAFLD) and alcohol abuse are more common [24]. HBV infection is well manageable whereas HCV infection (before the DAA era) would have been progressive after resection or destruction. NAFLD is associated with obesity and diabetes and both can potentially lead to serious comorbidity [25], and alcohol abuse may have continued or recurred. Differences in etiology of underlying liver disease or (their impact on) comorbidity as contributors to the observed survival disparities after resection could not be studied since these data were not present in our database.

Interestingly, no significant ethnical survival difference was observed after liver transplantation. These results do not match the study by Ananthakrishnan et al. [10], who reported that African American patients benefitted less from transplantation than Non-Hispanic Whites, using a United Network for Organ Sharing database [10]. However, our findings are in line with the data reported by Mathur et al [9], who also studied the SEER-database [9]. Therefore, these inconsistencies may be due to different patient populations analyzed. Although, Artinyan et al also used SEER data to report poorer survival after transplantation for African Americans [3], we believe that the population described in this particular study which included patients with diagnostic year as early as 1973 until 2004, is significantly different from our study population since implementation of the Milan criteria for liver transplantation in clinical practice only occurred after year of 1996 [26]. Therefore, differences in eligibility criteria for liver transplantation may explain the differences in results between our and their study.

We next explored a potential role for receiving liver transplantation on ethnical survival disparity. This issue has been discussed extensively over the years [6–8]. Several studies have demonstrated limited access to transplantation for African American patients, and indeed also in our study African Americans eligible for liver transplantation received this potentially curative treatment less frequently than non-Hispanic Whites. But as also reported before, so did Asian HCC patients [8–10, 12, 27–30]. Some studies have suggested that disparity in receiving transplantation may have contributed to ethnical disparity in survival [4, 9]. To determine the impact of undergoing liver transplantation on ethnical differences in survival, we analyzed the subgroup of patients who met the Milan criteria. Compared to Non-Hispanic Whites, Hispanic White and African American patients exhibited a poorer survival, and indeed their survival discrepancy disappeared after adjusting transplantation status. Asians “within Milan” on the other hand have better outcome which is unaffected by the factor liver transplantation. These patients have been shown to receive resection more often than liver transplantation and more often than any other race [7], and this is confirmed in our study. Since most of the Asian cases are likely HBV related [24] and may therefore have relatively preserved underlying liver function, more Asians can tolerate liver resection. It probably explains the small impact of transplantation on their survival. Of note, we could not identify a significant contributing role for socioeconomic or demographic factors to the ethnic survival discrepancy in this subgroup, suggesting that these factors may not determine access to transplantation. Indeed, whether or not to transplant is a complex decision making process that involves evaluation of etiology of liver disease, comorbidity, social context etc. and as said, these factors could not all be analyzed in our study.

Our work has some limitations. Firstly, since our study is retrospective in nature, it holds the known biases associated with this type of study. Secondly, as mentioned the level of clinical detail available to us does not capture significant details that may affect the use of surgical therapy or survival, such as medical comorbidities, presence of chronic liver disease and its etiology, and information on the details of all treatments received. Thirdly, the county-level socioeconomic data may not fully capture the economic, educational, and social factors for individual patients. Lack of social support, density of specialists within a region, hospital volume, distance to care, and other unmeasured confounders may have influenced access to therapies. Lastly, the effects of sorafenib or TACE on ethnical survival difference could not be studied since SEER has no specific coding for these treatment modalities. Nevertheless, to our knowledge, our analysis represents the most comprehensive study on ethnic differences in survival for HCC patients in the US.

In conclusion, we have confirmed the ethnical disparities in overall survival of HCC patients in the US. Poor tumor-presentation at diagnosis, poor response to resection, and limited utilization of transplantation all play essential roles in the poorer survival of African Americans compared to other races. Asian patients have superior survival, but after liver transplantation ethnic disparity in survival is absent.

## MATERIALS AND METHODS

### Patients selection

This study was performed using data from the Surveillance, Epidemiology, and End Results (SEER) Program (www.seer.cancer.gov) SEER*Stat Database (version 8.2.1). The procedure for selecting the patients for the cohort is shown in Supplementary Figure 1. Briefly, these patients were diagnosed between 2004 and 2012. We included the following ethnicities: Non-Hispanic White, Hispanic White, African American, and Asian (Asian/Pacific Islander). Among the Asian population in this study, 41.4% (2360/5699) were East Asians, 3.8% (219/5699) were South Asians, 39.2% (2233/5699) were Southeast Asians, and 15.6% were other Asians (887/5699). Native American (American Indian/Alska native) were excluded from our study. SEER Vital status recode (study cutoff used) variable was used to define the status of patients after the last follow-up date: death and alive. The survival time months variable, starting from diagnosis to last follow-up, was used for extracting information on patients’ survival time. The follow-up cut-off date was December 31, 2012. Among the overall population, we selected patients within Milan criteria: one lesion ≤ 5 cm or up to 3 lesions each with diameter ≤ 3 cm; no extra-hepatic involvement; and no vascular invasion.

### Definition

SEER Staging (also called Summary Staging) was used to define HCC stage: localized, regional, and distant. SEER Staging is the most basic way of categorizing how far a cancer has spread from its point of origin, as it combines the most precise clinical and pathological documentation of the extent of disease (http://training.seer.cancer.gov/ss2k/staging/). The detailed SEER Staging for HCC is documented in the “SEER Summary Staging Manual”. For example, localized HCC indicates the cancer confined to one lobe with or without vascular invasion, or multiple nodules/tumors confined to one lobe.

HCC therapies were categorized into groups based on data available in SEER database: none, local tumor destruction, surgical resection, and liver transplantation (LT). None indicated: without any intervention such as local tumor destruction, surgical resection, or liver transplantation. Local tumor destruction included: photodynamic therapy (PDT), electrocautery, cryosurgery, laser, PEI, heat-radio-frequency ablation (RFA). Since SEER has no specific coding for chemotherapy (sorafenib) or chemoembolization (TACE), they were not specified as such in SEER database. Resection included wedge, segmental resection, and lobectomy. “Unknown” means uncertainty about whether surgery was performed or what type of surgery was done.

The following SES related variables were included: education (the percentage of adults aged ≥ 25 years who < 12 years of education), poverty (the percentage of individuals living below the poverty line), and income (median annual household income). These variables were used as continuous variables in this study. According to the definitions of Country Attributes in SEER data, the higher values of the variables of education and poverty are, the lower the values of SES are. Please see “ http://seer.cancer.gov/seerstat/variables/countyattribs/#08–12” for details.

### Statistical analysis

Descriptive statistics were reported as both mean with standardized deviation (SD), and median with interquartile range (IQR) for continuous variables, and whole number with percentage for categorical variables. One-way ANOVA was used to compare groups for continuous variables. Pearson Chi-Square was used for comparing groups for categorical variables. Crude (non-adjustment) survival analysis (Kaplan-Meier curve) was first used to display the overall observed ethnical survival differences. Hazard ratio (HRs) and 95% CIs were calculated to evaluate the prognostic power of variables in survival. The included variables were divided into three categories:1) demographic variables, including race, age, SEER site, gender, marital status, education, income, poverty, residence; 2) presentation-related variables, including grade, stage, number of lesion, tumor size, lymph node involvement (yes or no), vascular invasion (yes or no), metastatic status (yes or no), AFP (Alpha-fetoprotein), and fibrosis degree (none to moderate fibrosis; several fibrosis or cirrhosis); and 3) treatment-related variables, including treatment presenting with categories — no treatment, tumor destruction, resection, and transplantation. For the procedure of multivariate cox model, race was entered as block 1, the remaining demographic variables were entered as block 2 (including age, gender, marital status, education, income, poverty, residence), presentation-related variables were entered as block 3 (including tumor size, stage, grade, number of lesion, lymph node status, vascular invasion status, metastatic status, AFP, and fibrosis staging), and finally treatment-related variable was entered as block 4. All analysis were stratified on SEER site to adjust for any heterogeneity between sites. Regarding block 2 and 3, the covariates were entered in a forward stepwise manner using the Likelihood Ratio test (LR) to describe their impact on survival. The changes in the HR's of African Americans, Hispanic White and Asian versus Non-Hispanic White after the stepwise adjustment of the covariates are shown in forest plots. There were some missing values for several categorical variables in our study. As we did not find any significant differences from the analysis of all cases and the cases with known values, we treated the missing values (with “unknown” label shown in Tables 1, 2) as a separate subcategory. Data preparation and forest plot were done in R (version 3.3.1). Statistical analyses was performed in SPSS (version 21); syntax shown in Supplementary Figure 2. *P* < .05 (two tailed sides) was considered as significant.

## SUPPLEMENTARY MATERIALS FIGURES AND TABLES





## References

[1] Mittal S, El-Serag HB (2013). Epidemiology of hepatocellular carcinoma: consider the population. J Clin Gastroenterol.

[2] Wang S, Sun H, Xie Z, Li J, Hong G, Li D, Mallampati S, Zhou X, Zhou C, Zhang H, Cheng Z, Shan H, Ma H (2016). Improved survival of patients with hepatocellular carcinoma and disparities by age, race, and socioeconomic status by decade, 1983–2012. Oncotarget.

[3] Artinyan A, Mailey B, Sanchez-Luege N, Khalili J, Sun CL, Bhatia S, Wagman LD, Nissen N, Colquhoun SD, Kim J (2010). Race, ethnicity, and socioeconomic status influence the survival of patients with hepatocellular carcinoma in the United States. Cancer.

[4] Xu L, Kim Y, Spolverato G, Gani F, Pawlik TM (2016). Racial disparities in treatment and survival of patients with hepatocellular carcinoma in the United States. Hepatobiliary Surg Nutr.

[5] Davila JA, El-Serag HB (2006). Racial differences in survival of hepatocellular carcinoma in the United States: a population-based study. Clin Gastroenterol Hepatol.

[6] Sloane D, Chen H, Howell C (2006). Racial disparity in primary hepatocellular carcinoma: tumor stage at presentation, surgical treatment and survival. J Natl Med Assoc.

[7] Ha J, Yan M, Aguilar M, Tana M, Liu B, Frenette CT, Bhuket T, Wong RJ (2016). Race/Ethnicity-specific Disparities in Hepatocellular Carcinoma Stage at Diagnosis and its Impact on Receipt of Curative Therapies. J Clin Gastroenterol 2015.

[8] Siegel AB, McBride RB, El-Serag HB, Hershman DL, Brown RS, Renz JF, Emond J, Neugut AI (2008). Racial disparities in utilization of liver transplantation for hepatocellular carcinoma in the United States, 1998–2002. Am J Gastroenterol.

[9] Mathur AK, Osborne NH, Lynch RJ, Ghaferi AA, Dimick JB, Sonnenday CJ (2010). Racial/ethnic disparities in access to care and survival for patients with early-stage hepatocellular carcinoma. Arch Surg.

[10] Ananthakrishnan AN, Saeian K (2008). Racial differences in liver transplantation outcomes in the MELD era. Am J Gastroenterol.

[11] Ward E, Jemal A, Cokkinides V, Singh GK, Cardinez C, Ghafoor A, Thun M (2004). Cancer disparities by race/ethnicity and socioeconomic status. CA Cancer J Clin.

[12] Hoehn RS, Hanseman DJ, Wima K, Ertel AE, Paquette IM, Abbott DE, Shah SA (2015). Does race affect management and survival in hepatocellular carcinoma in the United States?. Surgery.

[13] Altekruse SF, Henley SJ, Cucinelli JE, McGlynn KA (2014). Changing hepatocellular carcinoma incidence and liver cancer mortality rates in the United States. Am J Gastroenterol.

[14] Ha J, Yan M, Aguilar M, Bhuket T, Tana MM, Liu B, Gish RG, Wong RJ Race/ethnicity-specific disparities in cancer incidence, burden of disease, and overall survival among patients with hepatocellular carcinoma in the United States LID.

[15] Alawadi ZM, Phatak UR, Kao LS, Ko TC, Wray CJ (2016). Race not rural residency is predictive of surgical treatment for hepatocellular carcinoma: Analysis of the Texas Cancer Registry. Journal of Surgical Oncology.

[16] Wong RJ, Devaki P, Nguyen L, Cheung R, Nguyen MH (2014). Ethnic disparities and liver transplantation rates in hepatocellular carcinoma patients in the recent era: results from the Surveillance, Epidemiology, and End Results registry. Liver Transpl.

[17] Edge SB (2010). American Joint Committee on C. AJCC cancer staging manual.

[18] European Association For The Study Of The L European Organisation For R, Treatment Of C (2012). EASL-EORTC clinical practice guidelines: management of hepatocellular carcinoma. J Hepatol.

[19] Yau T, Tang VY, Yao TJ, Fan ST, Lo CM, Poon RT (2014). Development of Hong Kong Liver Cancer staging system with treatment stratification for patients with hepatocellular carcinoma. Gastroenterology.

[20] Wang L, Wilson SE, Stewart DB, Hollenbeak CS (2011). Marital status and colon cancer outcomes in US Surveillance, Epidemiology and End Results registries: does marriage affect cancer survival by gender and stage?. Cancer Epidemiol.

[21] Martinez ME, Anderson K, Murphy JD, Hurley S, Canchola AJ, Keegan TH, Cheng I, Clarke CA, Glaser SL, Gomez SL (2016). Differences in marital status and mortality by race/ethnicity and nativity among California cancer patients. Cancer.

[22] Gomez SL, Hurley S, Canchola AJ, Keegan TH, Cheng I, Murphy JD, Clarke CA, Glaser SL, Martinez ME (2016). Effects of marital status and economic resources on survival after cancer: A population-based study. Cancer.

[23] Wang XD, Qian JJ, Bai DS, Li ZN, Jiang GQ, Yao J (2016). Marital status independently predicts pancreatic cancer survival in patients treated with surgical resection: an analysis of the SEER database. Oncotarget.

[24] Hwang SJ, Tong MJ, Lai PP, Ko ES, Co RL, Chien D, Kuo G (1996). Evaluation of hepatitis B and C viral markers: clinical significance in Asian and Caucasian patients with hepatocellular carcinoma in the United States of America. J Gastroenterol Hepatol.

[25] Prenner S, Rinella ME (2016). Moderate Exercise for Nonalcoholic Fatty Liver Disease. JAMA Intern Med.

[26] Mazzaferro V, Regalia E, Doci R, Andreola S, Pulvirenti A, Bozzetti F, Montalto F, Ammatuna M, Morabito A, Gennari L (1996). Liver transplantation for the treatment of small hepatocellular carcinomas in patients with cirrhosis. N Engl J Med.

[27] Zak Y, Rhoads KF, Visser BC (2011). Predictors of surgical intervention for hepatocellular carcinoma: race, socioeconomic status, and hospital type. Arch Surg.

[28] Freeman RB (2008). Liver transplantation for hepatocellular carcinoma: racial disparities?. Am J Gastroenterol.

[29] Sonnenday CJ, Dimick JB, Schulick RD, Choti MA (2007). Racial and geographic disparities in the utilization of surgical therapy for hepatocellular carcinoma. J Gastrointest Surg.

[30] Sarpel U, Suprun M, Sofianou A, Berger Y, Tedjasukmana A, Sekendiz Z, Bagiella E, Schwartz ME (2016). Disentangling the effects of race and socioeconomic factors on liver transplantation rates for hepatocellular carcinoma. Clin Transplant.

