# Supplementary material for: Factors associated with ethnical disparity in overall survival for patients with hepatocellular carcinoma
Source: Oncotarget. 2017 Jan 20;8(9):15193–204. doi: 10.18632/oncotarget.14771 (PMC5362478; doi:10.18632/oncotarget.14771)
Supplement: Supplementary file 1 [file oncotarget-08-15193-s001.pdf]

Factors associated with ethnical disparity in overall survival for patients with hepatocellular carcinoma

Supplementary Materials

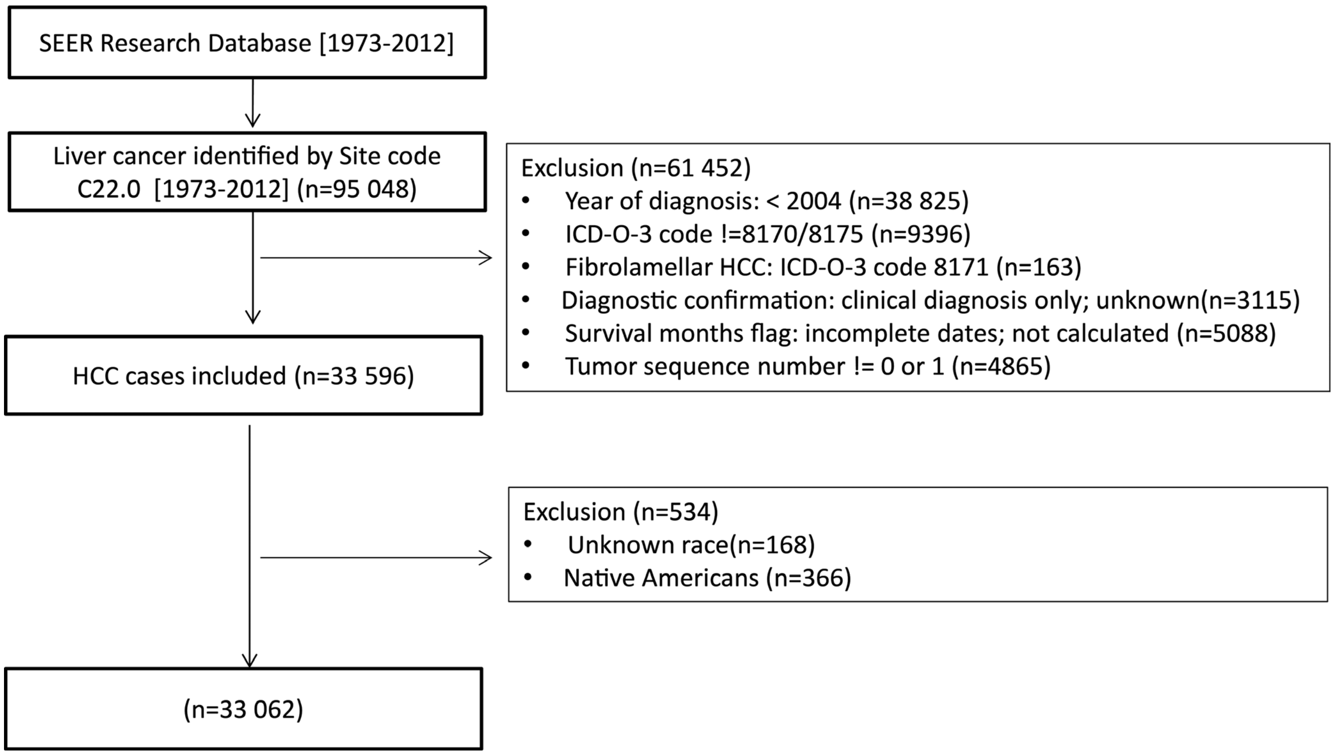

Supplementary Figure 1: Flowchart displaying the selection procedure of HCC cases in SEER database.

```
COXREG Time

/STATUS=Vital(1)

/STRATA SEER_Site (SEER site was stratified for all analysis)

/CONTRAST (Race)=INDICATOR(1)

/CONTRAST (Gender)=INDICATOR(1)

/CONTRAST (Marriage)=INDICATOR(1)

/CONTRAST (TumorSize)=INDICATOR(1)

/CONTRAST (Stage)=INDICATOR(1)

/CONTRAST (Grade)=INDICATOR(1)

/CONTRAST (LesionNumber)=INDICATOR(1)

/CONTRAST (LymphNode)=INDICATOR(1)

/CONTRAST (Vascular)=INDICATOR(1)

/CONTRAST (Metastasis)=INDICATOR(1)

/CONTRAST (AFP)=INDICATOR(1)

/CONTRAST (Fibrosis)=INDICATOR(1)

/CONTRAST (Surgery)=INDICATOR(1)

/METHOD=ENTER  Race

/METHOD=FSTEP(lr)   Age Gender Marriage Education Income Poverty Residence

/METHOD =FSTEP(lr) TumorSize Stage Grade LesionNumber  LymphNode  Vascular Metastasis AFP
Fibrosis

/METHOD=FSTEP(lr) Treatment

/PRINT=CI(95)

/CRITERIA=PIN(.05) POUT(.10) ITERATE(20).
```

Supplementary Figure 2: SPSS syntax for performing multivariate analysis in this study.

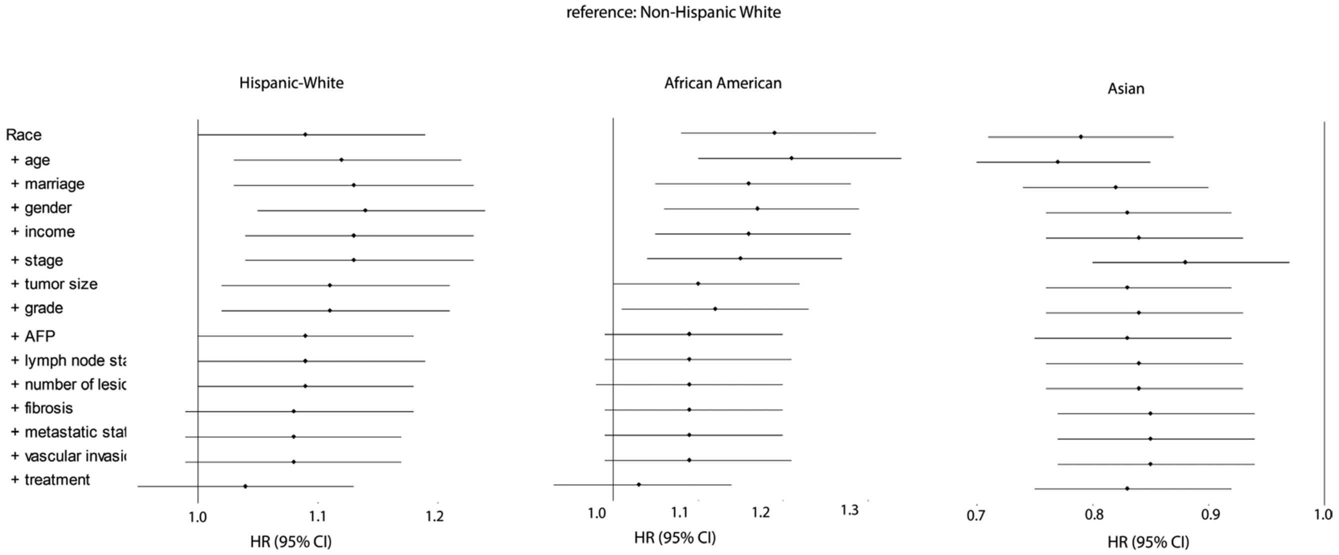

Supplementary Figure 3: Forest plot presenting the estimated HR's of ethnicity on overall survival from multivariate Cox models for all ethnical groups with available fibrosis score (reference: Non-Hispanic white). The first HR is the crude effect followed by HR after adjustment entering covariates in a forward stepwise manner (LR) : age, marriage, gender, education, grade, tumor size, AFP, fibrosis, lymph node status, number of lesion, and transplantation. Block 1 included race, block 2 included age, gender, marital status, education, income, poverty, residence, block 3 included grade, stage, number of lesion, tumor size, lymph node status, vascular invasion, metastatic status, AFP, and fibrosis, and block 4 included treatment.

Supplementary Table 1: Characteristics of the patients with known fibrosis value stratified by ethnicity. see Supplementary Table 1
