# Supplementary material for: Factors associated with ethnical disparity in overall survival for patients with hepatocellular carcinoma
Source: Oncotarget. 2017 Jan 20;8(9):15193–204. doi: 10.18632/oncotarget.14771 (PMC5362478; doi:10.18632/oncotarget.14771)
Supplement: Supplementary file 2 [file oncotarget-08-15193-s002.docx]

| **Supplemental Table S1:** Characteristics of the patients with known fibrosis value stratified by ethnicity. | | | | | |  |
| --- | --- | --- | --- | --- | --- | --- |
| **Characteristics** | **Total** | **Ethnicity** |  |  |  |  |
|  |  | **Non-Hispanic White** | **Hispanic White** | **African American** | **Asian** | ***P* Value** |
| Patients |  |  |  |  |  |  |
| No. % | 7070 | 3401 (48) | 1445(20) | 929 (13) | 1295(18) |  |
| Age |  |  |  |  |  | <.0001^a^ |
| Mean [SD],y | 61[10] | 61 [10] | 60 [10] | 60 [8] | 62 [12] |  |
| Median [IQR],y | 60[55-67] | 60 [55-67] | 59 [53-66] | 59 [55-64] | 62 [54-71] |  |
| Gender (%) |  |  |  |  |  | <.0001^b^ |
| Male | 5620 (80) | 2783 | 1141 | 723 | 973 |  |
| Female | 1450 (20) | 618 | 304 | 206 | 322 |  |
| Marital status (%) |  |  |  |  |  | <.0001^a^ |
| Married | 3723 (53) | 1743 | 747 | 318 | 915 |  |
| Unmarried | 3153 (45) | 1574 | 655 | 578 | 346 |  |
| Unknown | 194(3) | 84 | 43 | 33 | 34 |  |
| Education (%) |  |  |  |  |  | <.0001^b^ |
| Mean [SD] | 16 [6] | 15 [6] | 18 [7] | 15 [5] | 15 [5] |  |
| Median [IQR] | 14 [12-20] | 14 [11-18] | 16 [13-23] | 15 [12-16] | 14 [12-23] |  |
| Poverty (%) |  |  |  |  |  | <.0001^a^ |
| Mean [SD] | 15 [5] | 15 [5] | 16[5] | 18 [5] | 13 [4] |  |
| Median [IQR] | 14[12-20] | 14 [12-18] | 16 [13-18] | 18 [13-23] | 13 [10 - 14] |  |
| Income (%) |  |  |  |  |  | <.0001^a^ |
| Mean [SD] | 61885 [14989] | 59674 [14246] | 62087 [14426] | 55815 [13994] | 71822 [13537] |  |
| Median [IQR] | 59720 [53380-72760] | 57650 [49058-67180] | 56530 [54090-72110] | 55100 [41180 -6390] | 72760 [60450 -78760] |  |
| Residence (%) |  |  |  |  |  | <.0001^b^ |
| Rural | 469 (7) | 370 | 48 | 35 | 16 |  |
| Urban | 6601 (93) | 3031 | 1397 | 894 | 1279 |  |
| Lesion number (%) |  |  |  |  |  | .091^b^ |
| Single | 6822 (97) | 3264 | 1407 | 900 | 1251 |  |
| Multiple | 248 (4) | 137 | 38 | 29 | 44 |  |
| Grade (%) |  |  |  |  |  | <.0001^b^ |
| Well differentiated | 1017 (14) | 532 | 188 | 135 | 162 |  |
| Moderately differentiated | 1284 (18) | 625 | 222 | 176 | 261 |  |
| Poorly differentiated | 503 (7) | 223 | 85 | 62 | 133 |  |
| Undifferentiated | 45 (1) | 18 | 4 | 4 | 19 |  |
| Unknown | 4221 (60) | 2003 | 946 | 552 | 720 |  |
| Stage (%) |  |  |  |  |  | .013^b^ |
| Localized | 4066 (58) | 1947 | 822 | 511 | 786 |  |
| Regional | 2163 (31) | 1059 | 442 | 279 | 383 |  |
| Distant | 715 (10) | 327 | 158 | 122 | 108 |  |
| Unstaged | 126 (2) | 68 | 23 | 17 | 18 |  |
| Tumor size (cm),% |  |  |  |  |  | <.0001^b^ |
| <3 | 2123 (30) | 1091 | 421 | 255 | 356 |  |
| 3-5 | 2034 (29) | 965 | 424 | 262 | 383 |  |
| >5 | 2159 (31) | 955 | 450 | 303 | 451 |  |
| Unknown | 754 (11) | 390 | 150 | 109 | 105 |  |
| Lymph node involvement (%) |  |  |  |  |  | .010^b^ |
| No lymph node | 6822 (88) | 2955 | 1270 | 809 | 1168 |  |
| Lymph node | 390 (6) | 215 | 69 | 58 | 48 |  |
| Unknown | 478 (7) | 231 | 106 | 62 | 79 |  |
| Vascular Invasion (%) |  |  |  |  |  | .235^b^ |
| No Vascular Invasion | 3945 (56) | 1914 | 802 | 506 | 723 |  |
| Vascular Invasion | 2948 (42) | 1392 | 612 | 394 | 550 |  |
| Unknown | 177 (3) | 95 | 31 | 29 | 22 |  |
| Metastatic status (%) |  |  |  |  |  | .002^b^ |
| No metastasis | 6122 (86) | 2941 | 1238 | 787 | 1146 |  |
| Metastasis | 708 (10) | 326 | 158 | 120 | 104 |  |
| Unknown | 250 (4) | 134 | 49 | 22 | 45 |  |
| AFP^c^ (%) |  |  |  |  |  | <.0001^b^ |
| Positive | 4552 (64) | 2061 | 968 | 678 | 845 |  |
| Negative | 1545 (22) | 821 | 289 | 137 | 298 |  |
| Borderline | 17 (0.2) | 10 | 2 | 2 | 3 |  |
| Unknown | 956 (14) | 509 | 186 | 112 | 149 |  |
| Fibrosis (%) |  |  |  |  |  | <.0001^b^ |
| None to moderate fibrosis | 1622 (23) | 723 | 236 | 220 | 443 |  |
| Severe fibrosis or cirrhosis | 5448 (77) | 2678 | 1209 | 709 | 852 |  |
| Therapy (%) |  |  |  |  |  | <.0001^b^ |
| None | 4590 (65) | 2172 | 1052 | 651 | 715 |  |
| Tumor destruction | 787 (11) | 391 | 141 | 96 | 159 |  |
| Surgical resection | 889 (13) | 374 | 89 | 117 | 309 |  |
| Liver transplantation | 773 (11) | 443 | 159 | 61 | 110 |  |
| Unknown | 31 (0.4) | 21 | 4 | 4 | 2 |  |
| ^a^ one-way ANOVA test  ^b^ Pearson Chi-Square  ^c^ AFP positive indicates AFP >15 g/ml. |  | | | | | |
